# Supplementary material for: Rebound in functional distinctiveness following warming and reduced fishing in the North Sea
Source: Proc Biol Sci. 2021 Jan 13;288(1942):20201600. doi: 10.1098/rspb.2020.1600 (PMC7892419; doi:10.1098/rspb.2020.1600)
Supplement: Data Sources; Supplementary tables; Supplementary figures [file rspb20201600supp1.pdf]

Supplementary material

Paper details:

- Authors: Juliette Murgier, Matthew McLean, Anthony Maire, David Mouillot, Nicolas Loiseau, François Munoz, Cyrille Violle & Arnaud Auber

- Title: Rebound in functional distinctiveness following warming and reduced fishing in the North Sea

- Journal name: Proceedings of the Royal Society B

- DOI: 10.1098/rspb.[paper ID in form xxxx.xxxx e.g. 10.1098/rspb.2016.0049].

- ID: RSPB-2020-1600.R1

Appendix 1 – Data Sources

Froese, R., Pauly, D., 2018. FishBase (www database). < http://www.Fishbase.org >

Global Biodiversity Information Facility. <https://www.gbif.org/>

ICES (International Council for the Exploration of the Sea), 2018. Greater North Sea Ecoregion-Fisheries Overview. < https://doi.org/10.17895/ices.pub.4647 >

IUCN 2015. IUCN Red List of Threatened Species. Version 2015-4 < www.iucnredlist.org >

National Oceanic and Atmospheric Administration (NOAA, US), NAO values. <http://www.cpc.ncep.noaa.gov/products/precip/CWlink/pna/nao_index.html >

National Oceanic and Atmospheric Administration (NOAA, US), 2014. AMO values. <http://www.cdc.noaa.gov/Timeseries/>

Ocean Biogeographic Information System <www.iobis.org/>

SAHFOS (Sir Alister Hardy Foundation for Ocean Science), 2016. Phytoplankton Colour Index data from the North Sea from 1958-2014 provided by SAHFOS. < https://www.cprsurvey.org/>

Sir Alister Hardy Foundation for Ocean Science (SAHFOS) Continuous Plankton Recorder Dataset (SAHFOS). < https://doi.org/10.7487/2017.257.1.1081 >

SMS (Stochastic Multi Species model) < http://ices.dk/community/groups/Pages/WGSAM.aspx >

Appendix 2 – Supplementary tables

Table S1. Ecological traits of North Sea fish species considered in this study along with the reasoning and references for choosing each trait. Adapted from McLean et al. (2019).

Category	Trait	Reasoning	Type	Attributes	References
Habitat preference	Position in the water column	Influences distribution, dispersal, mobility.	Categorical	Demersal<br>Pelagic<br>Benthopelagic<br>Reefassociated	Alheit et al., 2014<br>MonteroSerra et al., 2014<br>Rijnsdorp et al., 2009
Trophic ecology	Trophic level	Influences position within the food web, impacts on carbon and nutrient fluxes.	Numeric	Continuous<br>Minimum: 2.2<br>Maximum: 4.5	Hempson et al., 2018<br>Huxel and McCann, 1998<br>Schneider et al., 2016
	Diet	Influences distribution, population growth rate, population size, impacts on carbon and nutrient fluxes.	Categorical	Benthivorous<br>Benthopiscivorous<br>Carcinophageous<br>Detritivorous<br>Ectoparasite<br>Piscivorous<br>Planktivorous<br>Scavenger	Albouy et al., 2011<br>Finke and Denno, 2005
Life-history	Age at sexual maturity	Influences growth rate, speed of maturation and reproduction, population turnover.	Numeric	Continuous<br>Minimum: 0.33 year<br>Maximum: 15 years	Crozier and Hutchings, 2014<br>King and McFarlane, 2003<br>Mims and Olden, 2012<br>Pankhurst and Munday, 2011
	Size at sexual maturity	Influences growth rate, metabolism, feeding rate, mobility, position in the food web.	Numeric	Continuous<br>Minimum: 2.65 cm<br>Maximum: 125.3 cm	Brown et al., 2004<br>Fisher et al., 2010<br>Petchey et al., 2008
	Fecundity (offspring number)	Influences population growth rate, dispersal rate, population turnover.	Numeric	Continuous<br>Minimum: 2 offsprings<br>Maximum: 9 106 offsprings	Lambert, 2008<br>Pécuchet et al., 2017<br>Pörtner et al., 2001
	Offspring size	Determines offspring survival and dispersal.	Numeric	Continuous<br>Minimum: 0.34 cm<br>Maximum: 345 cm	Adams, 1980<br>Pianka, 1970<br>Sirot et al., 2015<br>Ware, 1975
	Investment in parental care	Determines offspring survival and dispersal. Represents an energetic trade-off in life-history strategy.	Categorical	1: pelagic eggs<br>2: benthic eggs<br>3: hidden brood<br>4: protected brood<br>5: live bearing	Smith and Wootton, 1995<br>Winemiller and Rose, 1992

Adams, P. B. (1980). Life history patterns in marine fishes and their consequences for fisheries management. Fishery bulletin, 78(1), 1-12.

Albouy, C., Guilhaumon, F., Villéger, S., Mouchet, M., Mercier, L., Culioli, J. M., & Mouillot, D. (2011). Predicting trophic guild and diet overlap from functional traits: statistics, opportunities and limitations for marine ecology. *Marine Ecology Progress Series*, *436*, 17-28.

Alheit, J., Licandro, P., Coombs, S., Garcia, A., Giráldez, A., Santamaría, M. T. G., & Tsikliras, A. C. (2014). Reprint of "Atlantic Multidecadal Oscillation (AMO) modulates dynamics of small pelagic fishes and ecosystem regime shifts in the eastern North and Central Atlantic". *Journal of Marine Systems*, *133*, 88-102.

Crozier, L. G., & Hutchings, J. A. (2014). Plastic and evolutionary responses to climate change in fish. *Evolutionary Applications*, *7*(1), 68-87.

Finke, D. L., & Denno, R. F. (2005). Predator diversity and the functioning of ecosystems: the role of intraguild predation in dampening trophic cascades. *Ecology letters*, *8*(12), 1299-1306.

Fischer, A. H., Henrich, T., & Arendt, D. (2010). The normal development of Platynereis dumerilii (Nereididae, Annelida). *Frontiers in zoology*, *7*(1), 31.

Hempson, T. N., Graham, N. A., MacNeil, M. A., Hoey, A. S., & Wilson, S. K. (2018). Ecosystem regime shifts disrupt trophic structure. *Ecological Applications*, *28*(1), 191-200.

Huxel, G. R., & McCann, K. (1998). Food web stability: the influence of trophic flows across habitats. *The american naturalist*, *152*(3), 460-469.

King, J. R., & McFarlane, G. A. (2003). Marine fish life history strategies: applications to fishery management. *Fisheries Management and Ecology, 10*(4), 249-264.

Lambert, Y. (2008). Why should we closely monitor fecundity in marine fish populations. *J. Northw. Atl. Fish. Sci, 41*, 93-106.

Mims, M. C., & Olden, J. D. (2012). Life history theory predicts fish assemblage response to hydrologic regimes. *Ecology*, *93*(1), 35-45.

Montero-Serra, I., Páez-Rosas, D., Murillo, J. C., Vegas-Vilarrúbia, T., Fietz, K., & Denkinger, J. (2014). Environment-driven changes in terrestrial habitat use and distribution of the Galapagos sea lion. *Endangered Species Research*, *24*(1), 9-19.

Pankhurst, N. W., & Munday, P. L. (2011). Effects of climate change on fish reproduction and early life history stages. *Marine and Freshwater Research*, *62*(9), 1015-1026.

Pecuchet, L., Lindegren, M., Hidalgo, M., Delgado, M., Esteban, A., Fock, H. O., & Payne, M. R. (2017). From traits to life-history strategies: Deconstructing fish community composition across European seas. *Global Ecology and Biogeography*, *26*(7), 812-822.

Petchey, O. L., Beckerman, A. P., Riede, J. O., & Warren, P. H. (2008). Size, foraging, and food web structure. *Proceedings of the National Academy of Sciences*, *105*(11), 4191-4196.

Pianka, E. R. (1970). On r-and K-selection. *The american Naturalist*, *104*(940), 592-597.

Pörtner, H. O., Berdal, B., Blust, R., Brix, O., Colosimo, A., De Wachter, B., & Lannig, G. (2001). Climate induced temperature effects on growth performance, fecundity and recruitment in marine fish: developing a hypothesis for cause and effect relationships in Atlantic cod (Gadus morhua) and common eelpout (Zoarces viviparus). *Continental Shelf Research*, *21*(18-19), 1975-1997.

Rijnsdorp, A. D., Peck, M. A., Engelhard, G. H., Möllmann, C., & Pinnegar, J. K. (2009). Resolving the effect of climate change on fish populations. *ICES journal of marine science*, *66*(7), 1570-1583.

Schneider, F. D., Brose, U., Rall, B. C., & Guill, C. (2016). Animal diversity and ecosystem functioning in dynamic food webs. *Nature Communications*, *7*(1), 1-8.

Sirot, C., Villéger, S., Mouillot, D., Darnaude, A. M., Ramos-Miranda, J., Flores-Hernandez, D., & Panfili, J. (2015). Combinations of biological attributes predict temporal dynamics of fish species in response to environmental changes. *Ecological Indicators*, *48*, 147-156.

Smith, C., & Wootton, R. J. (1995). The costs of parental care in teleost fishes. *Reviews in Fish Biology and Fisheries*, *5*(1), 7-22.

Ware, D. M. (1975). Relation between egg size, growth, and natural mortality of larval fish. *Journal of the Fisheries Board of Canada*, *32*(12), 2503-2512.

Winemiller, K. O., & Rose, K. A. (1992). Patterns of life-history diversification in North American fishes: implications for population regulation. *Canadian Journal of Fisheries and aquatic sciences*, *49*(10), 2196-2218.

Table S2: Summary of the redundancy analyses performed at quartile level. Explanatory variables considered are salinity 'SSS', shear stress 'Bstress', phytoplankton biomass 'PCI', sea surface temperature 'SST', trawling effort, North Atlantic Oscillation 'NAO' and Atlantic Multidecadal Oscillation 'AMO'.

**Question**	**Response variables**	**Explanatory variables**
**1**. Which variables explain the spatial distribution of functional distinctiveness?	Total abundance per ICES rectangle (33-year average values) for the species belonging to the first (Q1) and last (Q4) quartile of functional distinctiveness	33-year average values for each ICES rectangle:<br>SST<br>PCI<br>Salinity<br>Depth<br>Shear stress<br>Trawling effort
**2**. Which variables influence the temporal dynamics of functional distinctiveness?	Total abundance per year (average values of the 154 ICES rectangles for each year) for the species belonging to the first (Q1) and last (Q4) quartile of functional distinctiveness	Average values on the 154 ICES rectangles for each year:<br>SST<br>PCI<br>NAO<br>AMO<br>Salinity<br>Trawling effort
**3**. Are temporal trends in functional distinctiveness related to spatial environmental conditions?	Spearman correlation coefficient between the total abundance of the species belonging to the first (Q1) and last (Q4) quartile of functional distinctiveness and years per ICES rectangle.	33-year average values for each ICES rectangle:<br>SST<br>PCI<br>Salinity<br>Depth<br>Shear stress<br>Trawling effort

Table S3: List of North Sea fish species by distinctiveness quartile. The functional distinctiveness (Di) of each species is also indicated.

Functionally common species (Q1)		Q2 group		Q3 group		Functionally distinct species (Q4)	
Species	Di	Species	Di	Species	Di	Species	Di
Chelidonichthys lucerna	0.207	*Spondyliosoma cantharus*	0.235	*Rajella lintea*	0.279	*Hyperoplus lanceolatus*	0.332
Eutrigla gurnardus	0.208	*Lepidorhombus whiffiagonis*	0.244	*Raja undulata*	0.281	*Hippocampus hippocampus*	0.339
Enchelyopus cimbrius	0.209	*Gobius spp*	0.245	*Raja clavata*	0.282	*Clupea harengus*	0.34
Mullus surmuletus	0.209	*Pollachius pollachius*	0.248	*Phrynorhombus norvegicus*	0.287	*Sebastes viviparus*	0.342
Arnoglossus spp	0.21	*Hippoglossoides platessoides*	0.25	*Scophthalmus rhombus*	0.287	*Engraulis encrasicolus*	0.343
Chelidonichthys cuculus	0.211	*Lumpenus lampretaeformis*	0.251	*Echiichthys vipera*	0.289	*Zeus faber*	0.343
Gaidropsarus mediterraneus	0.213	*Merlangius merlangus*	0.252	*Pollachius virens*	0.29	*Argentina silus*	0.344
Triglops spp	0.213	*Merluccius merluccius*	0.257	*Zoarces viviparus*	0.291	*Trisopterus esmarkii*	0.345
Triglops murrayi	0.213	*Phycis blennoides*	0.259	*Myxine glutinosa*	0.294	*Sprattus sprattus*	0.346
Microstomus kitt	0.213	*Scorpaena scrofa*	0.26	*Capros aper*	0.294	*Etmopterus spinax*	0.347
Gaidropsarus spp	0.213	*Pholis gunnellus*	0.261	*Scophthalmus maximus*	0.295	*Petromyzon marinus*	0.351
Leptoclinus maculatus	0.214	*Leucoraja naevus*	0.261	*Myoxocephalus scorpius*	0.295	*Maurolicus muelleri*	0.353
Limanda limanda	0.215	*Gasterosteus aculeatus*	0.261	*Atherina presbyter*	0.301	*Scomberesox saurus*	0.357
Trisopterus minutus	0.215	*Leucoraja circularis*	0.268	*Anguilla anguilla*	0.302	*Gadiculus argenteus*	0.358
Trigloporus lastoviza	0.215	*Brosme brosme*	0.269	*Osmerus eperlanus*	0.303	*Ammodytes spp*	0.358
Pleuronectes platessa	0.215	*Leucoraja fullonica*	0.269	*Anarhichas lupus*	0.304	*Sardina pilchardus*	0.363
Trisopterus luscus	0.216	*Pomatoschistus spp*	0.271	*Trachurus trachurus*	0.305	*Labrus bergylta*	0.366
Microchirus variegatus	0.216	*Spinachia spinachia*	0.272	*Ctenolabrus rupestris*	0.307	*Amblyraja radiata*	0.369
Lycodes vahlii	0.216	*Zeugopterus spp*	0.273	*Molva molva*	0.307	*Cyclopterus lumpus*	0.375
Ciliata spp	0.217	*Lycodes spp*	0.273	*Scomber scombrus*	0.308	*Crystallogobius linearis*	0.381
Trigla lyra	0.219	*Raja montagui*	0.273	*Molva dypterygia*	0.314	*Helicolenus dactylopterus*	0.392
Artediellus atlanticus	0.219	*Trachinus draco*	0.274	*Chimaera monstrosa*	0.314	*Entelurus aequoreus*	0.393
Buglossidium luteum	0.219	*Lesueurigobius friesii*	0.274	*Dicentrarchus labrax*	0.317	*Lampetra fluviatilis*	0.393
Buglossidium spp	0.219	*Galeus melastomus*	0.274	*Echiodon drummondii*	0.32	*Dipturus batis*	0.393
Gaidropsarus vulgaris	0.219	*Anarhichas minor*	0.275	*Alosa spp*	0.32	*Symphodus melops*	0.397
Callionymus spp	0.22	*Scyliorhinus canicula*	0.275	*Melanogrammus aeglefinus*	0.32	*Aphia minuta*	0.399
Agonus cataphractus	0.22	*Zeugopterus regius*	0.277	*Syngnathus spp*	0.322	*Liza ramada*	0.399
Solea solea	0.221	*Gadus morhua*	0.277	*Salmo spp*	0.325	*Scyliorhinus stellaris*	0.414
Liparis liparis	0.221	*Lophius budegassa*	0.277	*Micromesistius poutassou*	0.328	*Brama brama*	0.447
Glyptocephalus cynoglossus	0.221	*Raja brachyura*	0.278	*Belone belone*	0.329	*Conger conger*	0.479
Platichthys flesus	0.222	*Lophius piscatorius*	0.279	*Argentina sphyraena*	0.33	*Mustelus spp*	0.505
Liparis montagui	0.224	*Zeugopterus punctatus*	0.279	*Nerophis ophidion*	0.33	*Squalus acanthias*	0.551
Raniceps raninus	0.232					*Galeorhinus galeus*	0.606

Appendix 3 – Supplementary figures

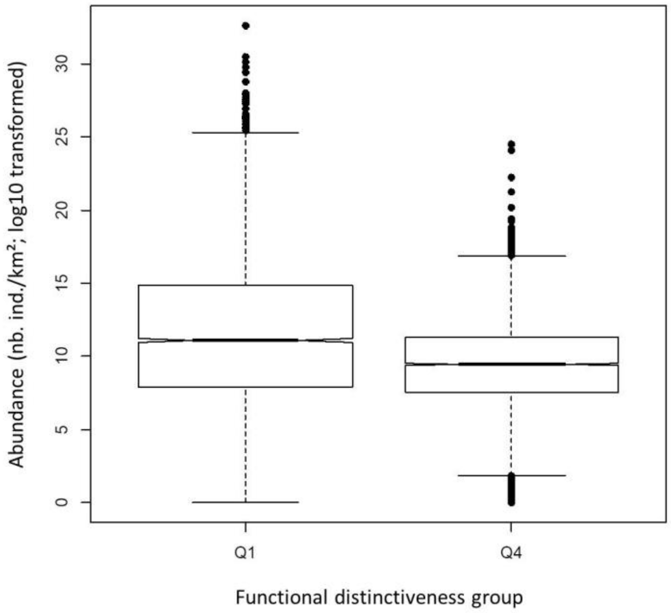

Figure S1. Boxplot showing the abundances of functionally common (Q1) and distinct (Q4) species. Each point corresponds to the abundance of a given species in a given ICES rectangle at a given year.

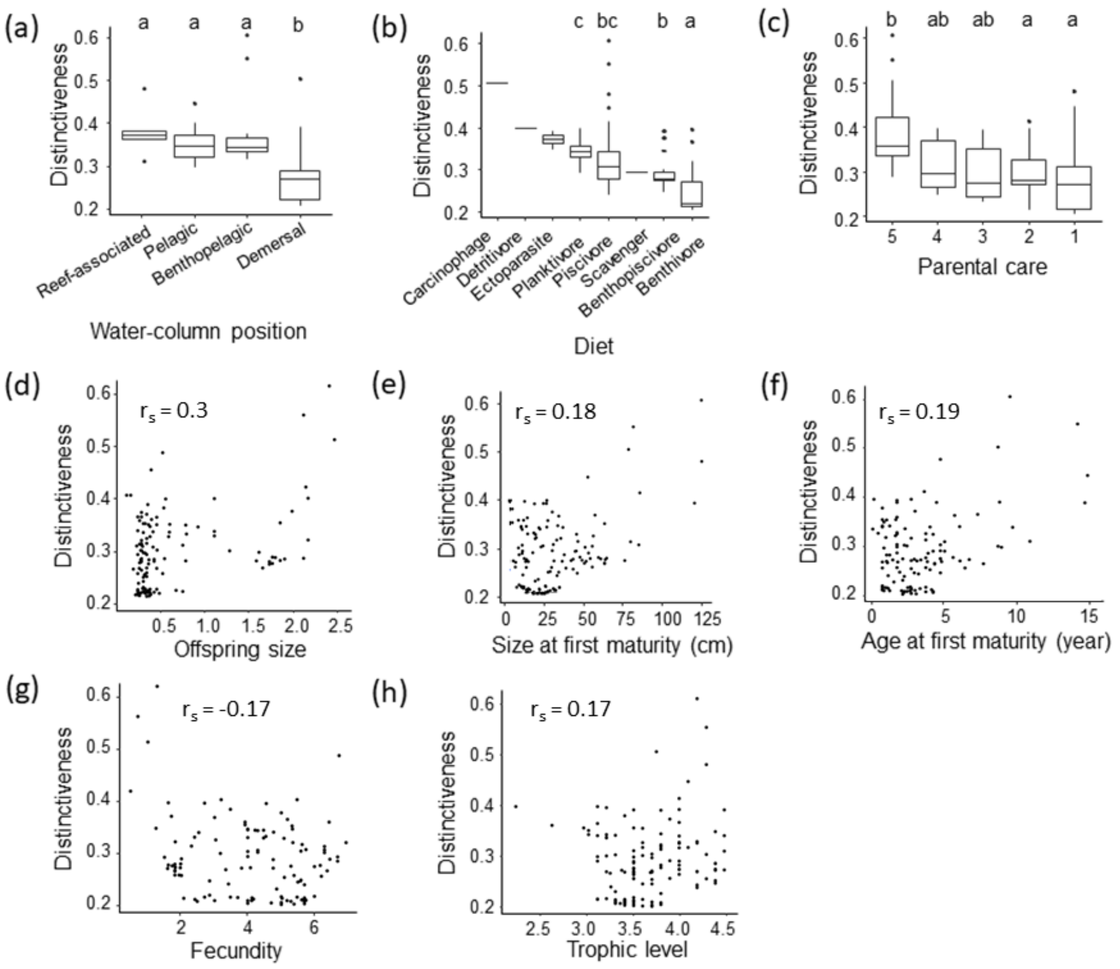

Figure S2. Statistical relationships between species' functional distinctiveness and ecological traits: (a) position in the water column, (b) diet, (c) investment in parental care, (d) offspring size (log10 transformed), (e) size at first maturity, (f) age at first maturity, (g) fecundity (log10 transformed), and (h) trophic level. For boxplots (panels a-c), different letters indicate significant differences between trait attributes (Wilcoxon post-hoc test).

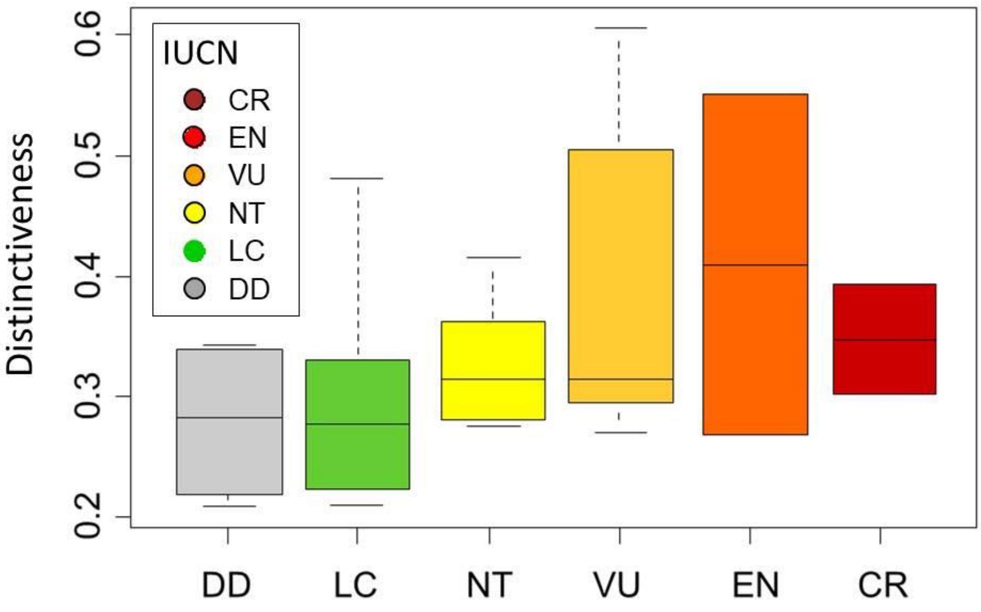

Figure S3. Statistical relationships between species' functional distinctiveness and IUCN status of species.

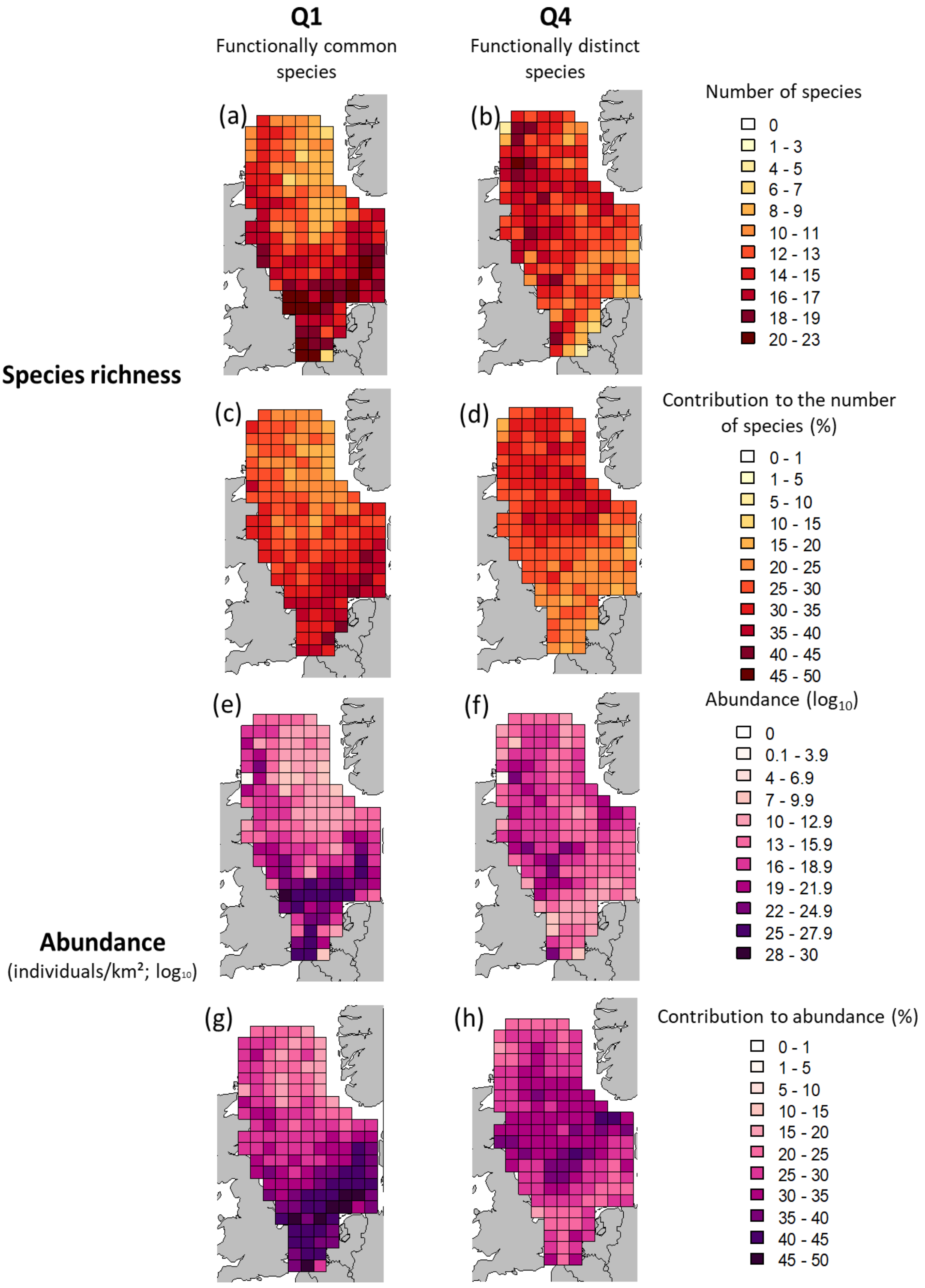

Figure S4. Spatial distribution of the functionally common (Q1; a, c, e and g) and distinct (Q4; b, d, f and h) species in the North Sea in terms of species richness (a and c) or total abundance (b and d) and spatial distribution of the contribution of functionally common and distinct species to the species richness (a and c) and total abundance (b and d) in each ICES rectangles.
